# The *wavy* Mutation Maps to the *Inositol 1,4,5-Trisphosphate 3-Kinase 2* (*IP3K2*) Gene of *Drosophila* and Interacts with *IP3R* to Affect Wing Development

**DOI:** 10.1534/g3.115.024307

**Published:** 2015-11-25

**Authors:** Derek M. Dean, Luana S. Maroja, Sarah Cottrill, Brent E. Bomkamp, Kathleen A. Westervelt, David L. Deitcher

**Affiliations:** * Department of Biology, Williams College, Williamstown, Massachusetts 01267; † Department of Neurobiology and Behavior, Cornell University, Ithaca, New York 14853

**Keywords:** Cam, GAL80, IP3K1, Ipk2, genetic interaction

## Abstract

Inositol 1,4,5-trisphosphate (IP_3_) regulates a host of biological processes from egg activation to cell death. When IP_3_-specific receptors (IP3Rs) bind to IP_3_, they release calcium from the ER into the cytoplasm, triggering a variety of cell type- and developmental stage-specific responses. Alternatively, inositol polyphosphate kinases can phosphorylate IP_3_; this limits IP3R activation by reducing IP_3_ levels, and also generates new signaling molecules altogether. These divergent pathways draw from the same IP_3_ pool yet cause very different cellular responses. Therefore, controlling the relative rates of IP3R activation *vs.* phosphorylation of IP_3_ is essential for proper cell functioning. Establishing a model system that sensitively reports the net output of IP_3_ signaling is crucial for identifying the controlling genes. Here we report that mutant alleles of *wavy* (*wy*), a classic locus of the fruit fly *Drosophila melanogaster*, map to *IP_3_ 3-kinase 2* (*IP3K2*), a member of the inositol polyphosphate kinase gene family. Mutations in *wy* disrupt wing structure in a highly specific pattern. RNAi experiments using GAL4 and GAL80^ts^ indicated that *IP3K2* function is required in the wing discs of early pupae for normal wing development. Gradations in the severity of the *wy* phenotype provide high-resolution readouts of *IP3K2* function and of overall IP_3_ signaling, giving this system strong potential as a model for further study of the IP_3_ signaling network. In proof of concept, a dominant modifier screen revealed that mutations in *IP3R* strongly suppress the *wy* phenotype, suggesting that the *wy* phenotype results from reduced IP_4_ levels, and/or excessive IP3R signaling.

The *Drosophila* wing has proven to be a reliable model for addressing research questions throughout the field of developmental biology. Its stereotyped, easily recognized, planar network of cuticular hairs and veins, external location, and dispensability for survival allow for efficient scoring of structural abnormalities, yet the tissue goes through a dynamic process to arrive at its adult form, providing opportunities to study several different developmental phenomena. Wing precursor cells are initially derived during early embryogenesis as epithelial cells on the ventrolateral margins of the second thoracic segment invaginate, forming common primordia for the wings and legs of that segment. Shortly thereafter, subsets of cells from these two primordia migrate dorsally to form a pair of wing imaginal discs (Cohen *et al.* 1993). Throughout the larval stages, cells of these imaginal discs undergo patterned proliferation, and positional information is integrated to specify the regions of the prospective wings and their vein boundaries (Milán *et al.* 1996a; Biehs *et al.* 1998; Klein 2001; Cavodeassi *et al.* 2002; Crozatier *et al.* 2002, 2004). Pupal stages are marked by eversion of the discs, an eventual cessation of cell proliferation, organization of epithelial cells into hexagonal arrays, the formation of a single cuticular hair at the distal vertex of each cell, and refinement of vein positions (Wong and Adler 1993; Milán *et al.* 1996b; Classen *et al.* 2005, 2008; Blair 2007; Taylor and Adler 2008). Shortly after adult eclosion, the wings, which remain folded during metamorphosis, are expanded by an increase in hemolymph pressure. At this point epithelial cells switch to a mesenchymal identity, delaminate from the overlying cuticular tissue, undergo programmed cell death, and are resorbed into the thorax (Kiger *et al.* 2007; Link *et al.* 2007; Natzle *et al.* 2008). During and after this process, the cuticular portions of the wing are left behind and intact, providing a clear readout of developmental perturbations that may have occurred anytime between embryogenesis and adult wing expansion. Therefore, the *Drosophila* wing acts as an accessible, one-stop destination to study a diverse array of cellular processes including fate determination, proliferation, morphogenesis, adhesion, polarity, migration, and programmed death.

The molecular genetic tractability of *Drosophila* has further facilitated inquiries into wing development. Researchers have employed a number of approaches successfully, including misexpression studies using wing-specific GAL4 drivers, RNAi, gene expression profiling of the developing wing, bioinformatics, and genetic interaction screens (Rorth *et al.* 1998; Ren *et al.* 2005; Du *et al.* 2011; Dui *et al.* 2012). These approaches have helped us better understand signal transduction networks that have been highly conserved throughout evolution [*e.g.*, wingless (Wnt), Notch (EGF), Hedgehog, and decapentaplegic (TGF-β)], as well as how these networks interact to affect development (Casso *et al.* 2011; Swarup and Verheyen 2012; Kwon *et al.* 2013; Yang *et al.* 2013; Hartl and Scott 2014).

Past studies have suggested that IP_3_ (inositol 1,4,5-trisphosphate) signaling could join the list of highly conserved signal transduction networks that are modeled by the developing *Drosophila* wing. A specific heteroallelic combination of mutations for the IP_3_ receptor gene *IP3R* has been reported to cause mild wing crumpling, and more combinations of *IP3R* alleles have been shown to affect wing “posture” (*i.e.*, the angle at which wings are held from the body), flight behavior, and the physiology of the flight circuit (Banerjee *et al.* 2004, 2006; Agrawal *et al.* 2009, 2010; Venkiteswaran and Hasan 2009). *IP3R* has been shown to act in the nervous system to affect wing posture and flight behavior/neurophysiology; however, the mechanism by which *IP3R* affects the morphology of the wing itself has remained unclear, and, to our knowledge, no other IP_3_ signaling genes have been reported to affect *Drosophila* wing development.

In eukaryotes, the ER lumen typically stores Ca^2+^, and IP3R is a Ca^2+^ channel located on the ER membrane. When IP_3_ binds to IP3R, the channel opens, releasing Ca^2+^ into the cytoplasm (Figure 1). This Ca^2+^ release goes on to affect many different cellular processes, including gamete activation, fertilization, proliferation, contraction, secretion, immune cell activation, and apoptosis (Xia and Yang 2005; Berridge 2009; Leanza *et al.* 2013; Ramos and Wessel 2013; Roderick and Knollmann 2013; Ambudkar 2014; Ivanova *et al.* 2014; Kaneuchi *et al.* 2015; Nohara *et al.* 2015; Shah *et al.* 2015; Vervloessem *et al.* 2015).

**Figure 1:**
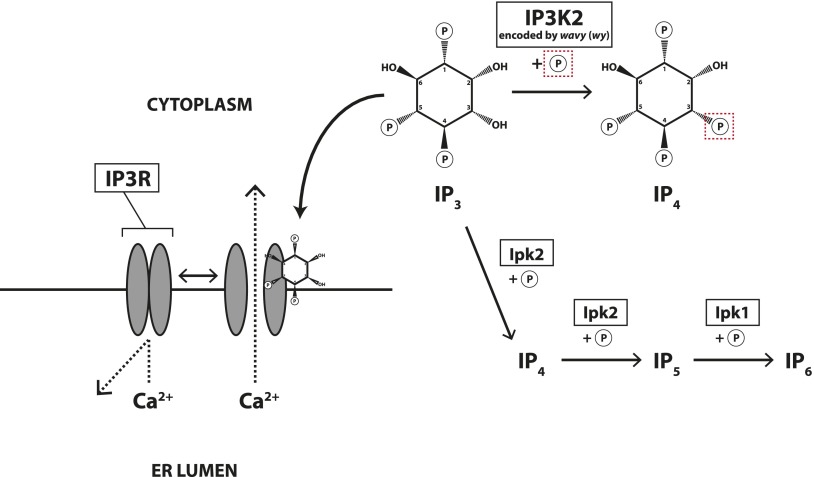
Some key components of IP_3_-related signaling in *Drosophila*. Enzyme names are boxed and an encircled “P” denotes an inorganic phosphate group. IP_3_ (inositol 1,4,5-trisphosphate, top center) may undergo the following fates: (1, left) bind to the IP_3_-gated calcium channel IP3R (IP3 receptor), causing IP3R to open and release calcium that was sequestered in the ER lumen, (2, top right) be phosphorylated by IP3K2 (IP_3_ 3-kinase 2) to form IP_4_ (inositol 1,3,4,5-tetrakisphosphate), or (3, bottom right) be more highly phosphorylated by Ipk2 (inositol polyphosphate kinase 2), and subsequently Ipk1 (inositol polyphosphate kinase 1) to yield IP_6_. Only forward reactions are shown because previous studies suggested that these reactions predominate in fly cells (Seeds *et al.* 2004). IP3K1 catalyzes the same reaction as IP3K2 but is not the primary focus of this study, and therefore not shown. Calmodulin (Cam), also not shown, increases IP3K2 activity by binding to the enzyme in a calcium-dependent fashion (Lloyd-Burton *et al.* 2007). See *Introduction* for additional molecular details. In this report, we present evidence that IP3K2 is encoded by the *wavy* (*wy*) locus, and that a balance between IP3K2 and IP3R functioning is necessary for normal wing morphology.

Alternatively, IP_3_ may be phosphorylated at the other positions around its hexagonal carbon ring, generating IP_4_, IP_5_, or IP_6_. In *Drosophila* (Figure 1), phosphorylation of IP_3_ can be accomplished by the IP3K2 enzyme (IP_3_ 3-kinase 2, the focus of this study), IP3K1 (IP_3_ 3-kinase 1), or by sequential action of the Ipk2 and Ipk1 enzymes (inositol polyphosphate kinases). IP3K2, IP3K1, and Ipk2 all convert IP_3_ to IP_4_ (inositol 1,3,4,5-tetrakisphosphate). IP3K2 may be affected by IP3R-mediated Ca^2+^ release from the ER: Calmodulin (Cam) binds to IP3K2 in a Ca^2+^-dependent manner, strongly upregulating IP3K2 activity (Seeds *et al.* 2004; Lloyd-Burton *et al.* 2007; Shah *et al.* 2015). However, unlike the IP3K enzymes of several other organisms that have been investigated, *Drosophila* IP3K2 and IP3K1 are not believed to provide IP_4_ for the synthesis of IP_5_ and IP_6_ (Seeds *et al.* 2004). Ipk2, on the other hand, can convert IP_3_ to one of two forms of IP_4_ (the aforementioned or phosphorylating at the 6′ instead of the 3′ position, yielding inositol 1,4,5,6-tetrakisphosphate), and then subsequently convert either IP_4_ isoform to IP_5_ (inositol 1,3,4,5,6-pentakisphosphate). Ipk1 then phosphorylates IP_5_ at the 2′ position, making IP_6_ (Seeds *et al.* 2004). In summary for *Drosophila*, possible fates for IP_3_ include: (1) its binding to IP3R to trigger the release of Ca^2+^ from the ER, (2) its phosphorylation by IP3K2 or IP3K1 to form IP_4_, or (3) its conversion to more highly phosphorylated species by the Ipk2/Ipk1 module (Figure 1).

Phosphorylation of IP_3_ has been shown to have functional consequences in eukaryotes. A number of proteins with IP_4_- or IP_6_-specific binding have been isolated (Donie *et al.* 1990; Theibert *et al.* 1991; Fukuda and Mikoshiba 1997; Xia and Yang 2005; Fain 2013). In mammals, IP_4_ appears to mediate Ca^2+^ transport into the intermembrane space of the nuclear envelope, as well as into the cytoplasm from outside the cell, and IP_6_ has been shown to act as a cofactor for enzymes involved in DNA damage repair, and RNA editing (Humbert *et al.* 1996; Hanakahi *et al.* 2000; Ma and Lieber 2002; Byrum *et al.* 2004; Macbeth *et al.* 2005; Xia and Yang 2005; Malviya and Klein 2006). The distinct receptors for—and functions of—the various inositol species suggest that coordinating their relative levels would be important in cellular functioning. *Drosophila* genetics provides an excellent toolbox to investigate this possibility, the *Drosophila* wing is a proven model system for investigating signal transduction in general, and wing morphology is at least mildly affected by IP3R function, suggesting that IP_3_ signaling plays a role in wing development (Banerjee *et al.* 2004).

Many classical mutations affecting the wings have not been mapped to their respective genes—this is presumably because a large number of *Drosophila* mutations, particularly viable alleles that affect external tissues such as the wing, were relatively easy to isolate and maintain in stocks long before molecular techniques were developed, creating a backlog. From such collections, we obtained a stock mutant for the *wavy* (*wy*) locus. Flies mutant for *wy* exhibit wings that are bent and crumpled in a highly specific pattern (Nachtsheim 1928; Parker 1935; Lindsley and Zimm 1992). Here we report the mapping of available *wy* alleles to *IP3K2* (*CG1630*). This gene encodes the IP_3_ 3-kinase 2 enzyme described above (Figure 1; Lindsley and Zimm 1992; Seeds *et al.* 2004; Lloyd-Burton *et al.* 2007). We also characterize the developmental window during which the *wy* function is required to specify wing morphology. Finally, we describe strong genetic interactions between *wy* and *IP3R*, suggesting a possible mechanism by which the IP_3_ signaling network affects wing morphology, *i.e.*, by balancing IP3R and IP3K2 activity. These findings help establish IP_3_ signaling as another highly conserved genetic network that is effectively modeled by the *Drosophila* wing.

## Materials and Methods

### wy alleles, mapping, complementation tests

The *wy^1^* (Bloomington Stock Center #168, or BL 168: *wy^1^*; Nachtsheim 1928), *wy^2^* (BL 192: *y^2^ wy^2^ g^2^*; Parker 1935), and *wy^74i^* alleles (BL 1294: *t^1^ v^1^ m^74i^ wy^74i^*; Lindsley and Zimm 1992) were used in this study. *wy^2^* was recombined into a *w f* background, and a *w wy^2^ f* stock was established to facilitate mapping and the tracking of *w^+^*-labeled constructs during crosses. *wy^2^*, *f*, and the *w^+^* transgene insertion lines PBac{WH}*CG12096^f05782^* (BL 18906: *w^1118^* Bac{WH}*CG12096^f05782^*), and P{EP}*Tango2^G517^* (BL32580: *w* P{EP}*Tango2^G517^*) were used for three-point recombination mapping (Bellen *et al.* 2004; Thibault *et al.* 2004). For complementation tests, *wy^1^*, *wy^2^*, or *wy^74i^* females were crossed to males carrying either of two duplications Dp(1;3)DC267 (BL 30384: *w^1118^*; Dp(1;3)DC267, PBac{DC267}VK00033) or Dp(1;3)DC268 (BL 30385: *w^1118^*; Dp(1;3)DC268, PBac{DC268}VK00033), and F1 *wy*/Y; Dp(1;3)/+ males were scored for the *wy* phenotype. In a second set of complementation tests, *wy^1^*, *wy^2^*, or *wy^74i^* males were crossed to females carrying either of two deletions [Df(1)BSC766 (BL 26863: Df(1)BSC766, *w^1118^*/Binsinscy) or Df(1)Exel6245 (BL 7718: Df(1)Exel6245, *w^1118^* P{XP-U}Exel6245/FM7c)], and F1 Df/*wy* females were scored for the *wy* phenotype (Parks *et al.* 2004; Venken *et al.* 2010; Cook *et al.* 2012).

### Phenotypic assessment and microscopy

A numerical scale was devised to quantify *wy* penetrance and expressivity throughout this study (see *Results* for a description of this scale). Wings were scored under a Leica dissecting scope, and photographs were taken using a NEX-3N-alpha camera body (Sony) attached to the microscope eyepiece with a T-Ring for Sony E Mount and 2-Inch Universal T Adapter (CNC Parts Supply, Inc.)

### PCR of *IP3K2* alleles and DNA sequencing

All chemicals were purchased from Sigma-Aldrich unless otherwise noted. Individual *wy^+^* (from a *y^1^ w^1^* strain, BL 1495), *wy^74i^*, and *wy^2^* adult male flies were first frozen in 1.5 ml Eppendorf tubes, then each was ground within their tube in 50 µl of standard fly “squishing” buffer [10 mM Tris (pH 8), 1 mM EDTA, 25 mM NaCl, 200 µg/ml Proteinase K]. Crushed flies were incubated for 30 min at 37° to digest fly tissue, then at 94° for 3 min to denature the Proteinase K. Segments of the *IP3K2* gene were PCR-amplified from DNA extract using GoTaq Flexi DNA polymerase (Promega; 1 µl DNA extract per 19 µl of standard reaction mix). PCR products were run through a 0.8% low-melt agarose gel to separate them from unincorporated primers, slabs containing the PCR products were excised from the gel, and products were purified from the agarose using the QIAquick Gel Extraction Kit (Qiagen). Purified PCR products were sent to the Cornell University Biotechnology Resource Center (Ithaca, NY) for sequencing using their recommended protocols. PCR/sequencing primers are described in Supporting Information, Table S1. Sequence outputs were analyzed using the MEGA5 software (Tamura *et al.* 2011). Sequences from at least two individual flies of each genotype were analyzed in order to resolve ambiguities.

### Rescue construct, RNAi of *IP3K2*, GAL4 driver, and GAL80^ts^

To assemble the rescue construct, a *Not*I–*Avr*II fragment containing the *IP3K2* open reading frame (restriction enzymes from New England Biolabs) was extracted from the RE35745 cDNA clone (GenBank accession number AY084158; Stapleton *et al.* 2002; Hoskins *et al.* 2011), ligated with T4 DNA ligase (New England Biolabs) into pUAS-c5-attB (Daniels *et al.* 2014) in order to place the *IP3K2* cDNA downstream of a UAS site, and finally sent to Bestgene (Chino Hills, CA) for transformation in a *w* background. Two independent insertion lines were obtained, both on the third chromosome at the 68A4 location. The manuscript refers to these rescue lines as UAS-*IP3K2*.

RNAi experiments of *IP3K2* were conducted with a stock from the Vienna Stock Center (VDRC v19159: P{GD8778}v19159/TM3 *Sb*), hereafter referred to as RNAi-*IP3K2* (Dietzl *et al.* 2007).

We used *nub*-GAL4 (BL 25754: P{UAS-*Dcr*-2.D}1, *w^1118^*; P{GawB}*nubbin*-AC-62), a wing disc-specific driver, for our RNAi experiments (Brand and Perrimon 1993; Azpiazu and Morata 2000). The *Tub*-GAL80^ts^ construct (from BL 7108: *w*; P{tubP-GAL80^ts^}10; TM2/TM6B, *Tb^1^*), which was employed for temperature-sensitive deactivation of *nub*-GAL4, was recombined onto the same chromosome as *nub*-GAL4, and a *w* stock was established that was also homozygous for both insertions but did not contain the P{UAS-*Dcr*-2.D}1 construct (also see *Fly culturing* below; Ferris *et al.* 2006; Baena-Lopez *et al.* 2009; Rodriguez *et al.* 2012).

### Dominant genetic modifier screen

We tested several IP_3_-signaling loci for genetic interactions with *wy*. The following alleles were obtained: (1) *IP3K1^KG02192^*, a *P*-insertion within an intron of *IP3K1*, (BL 14263: *y^1^*; P{SUPor-P}*IP3K1^KG02192^*/CyO; *ry^506^*; Bellen *et al.* 2004); (2) “Df-*Ipk2*“, a deletion spanning multiple genes including *Ipk2* [BL 9190: *w^1118^*; Df(2L)ED49/SM6a; Ryder *et al.* 2007]; (3) *Cam^n339^*, a deletion of *Cam* resulting from an imprecise *P*-element excision (BL 6806: *y^1^ w*; *Cam^n339^*/CyO, *y^+^*; Heiman *et al.* 1996), (4) *Cam^7^*, an EMS-induced point mutation (V91G) in the N-terminal helix region of the gene (BL 8140: *y^1^ w*; *J^1^ Cam^7^*/CyO, *y^+^*; Nelson *et al.* 1997); (5) *IP3R^90B.0^*, a deletion of the *IP3R* gene generated by an imprecise excision of a *P*-element (BL 30737: *IP3R^90B.0^*/TM6B *Tb^1^*; Venkatesh and Hasan 1997); and (6) *IP3R^ug3^*, an EMS-induced point mutation (S224F) in the IP_3_-binding domain (BL 30738: *IP3R^ug3^*/TM6B *Tb^1^*; Joshi *et al.* 2004).

### Fly culturing, and its modification for GAL4- and GAL80^ts^-based experiments

Flies were reared on a modified yeast/dextrose/cornmeal diet that is described in Supporting Information, File S1. Unless otherwise noted, culture maintenance and experimental conditions were at 25° under a 12h light:12h dark cycle in an incubator humidified to maintain conditions at 60–80% relative humidity.

Culturing was also modified for the GAL80^ts^ experiments. *nub*-GAL4 *Tub*-GAL80^ts^ females were mated to RNAi-*IP3K2* males, and vials containing the progeny from these crosses were incubated at either 18° to minimize expression of RNAi-*IP3K2*, or 29° to express RNAi-*IP3K2* at high levels (Ferris *et al.* 2006; Baena-Lopez *et al.* 2009; Rodriguez *et al.* 2012). Shifts from one temperature to the other were conducted at different developmental stages throughout the life cycle, and the wings of adult F1 flies were scored. A more detailed description of this experimental design is found in the *Results* section and in the Figure 4 caption.

### Data availability

The sequence assemblies for the *IP3K2* loci of *y^1^ w^1^*, *wy^2^*, and *wy^74i^* flies are deposited in GenBank under accession numbers KT732028, KT732029, and KT732027 respectively. The *w wy^2^ f* and *w*; UAS-*IP3K2* fly stocks and UAS-*IP3K2* construct are available upon request. All other fly stocks and reagents are commercially available.

## Results

### Characterization of the *wavy* (*wy*) phenotype

**Figure 2:**
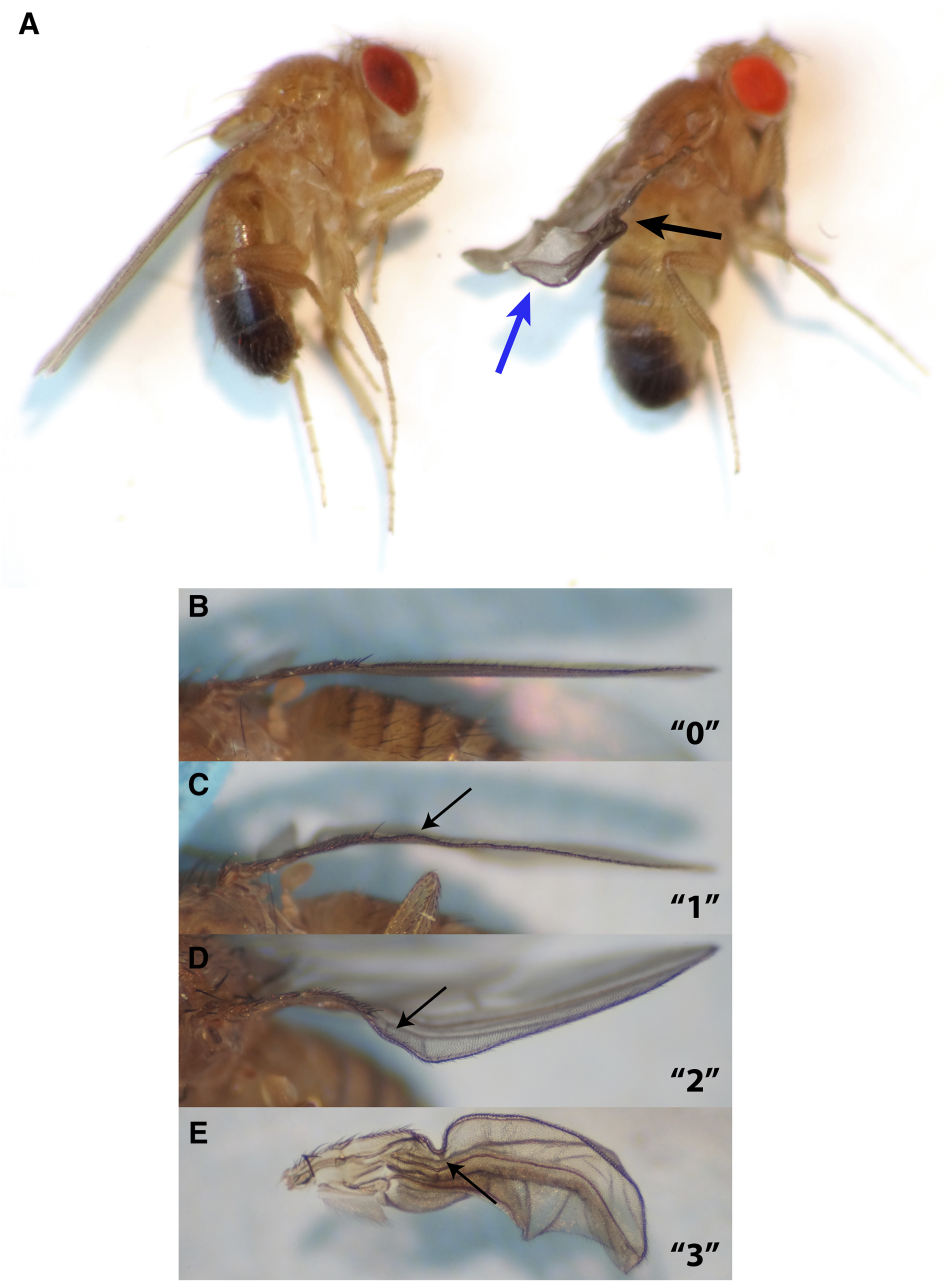
Mutations in *wavy* (*wy*) disrupted wing morphology. (A) Wild type (left) and *wy*^74i^ flies (right). Note that the *wy^74i^* flies obtained from the Bloomington Center (BL#1162) were also mutant for the eye color gene *vermillion* (*v^1^*). In the most extreme cases, a mutation in *wy* caused the following three phenotypes: (1) a wave-like buckle (black arrow) at a specific location midway along the costal vein, just distal to the intersection of this vein with the first longitudinal vein; (2) an upturn at the most distal margin of the wing (blue arrow); and (3) a generally shriveled appearance with a pattern. However, *wy* mutants often displayed only specific subsets of these phenotypes. (B–E) Numerical scoring system reflecting the subsets of *wy* phenotypes that were observed. (B) Phenotypically wild type wings received scores of “0” (note that none of the individuals from the original mutant strains received a wild type score; see Table 1A). (C) A wing with a costal buckle as the only apparent abnormality (black arrow) received a score of “1”. (D) A wing with a costal buckle and distal upturn was scored as a “2”. (E) A wing with a costal buckle, distal upturn, and overall shriveled appearance received a score of “3”. No other combinations of these three phenotypes were observed throughout our experiments.

We first examined the three classic mutant strains available from the Bloomington Center—*wy^1^*, *wy^2^*, and *wy^74i^*—to confirm and expand on their published phenotypic descriptions. As previously reported, the wings of *wy* mutants were severely deformed in a very specific pattern. In the most extreme cases, the wings of *wy* mutants exhibited all three of the following phenotypes (Figure 2A): (1) a wave-like buckle at a specific location along the costal vein, just distal to its intersection with the subcostal region; (2) an upturn at the most distal margin of the wing; and (3) an overall morphology that is shriveled but patterned in a manner that is readily distinguishable from nonspecific, mechanical wing damage, or from wings that fail to inflate after adult eclosion (example of failed inflation shown in Lahr *et al.* 2012). However, in many other cases, mutant flies exhibited a subset of these phenotypes, and, strikingly, only certain subsets were seen. We developed a numerical scale (0–3) to reflect the hierarchical pattern that we observed among the phenotypes, and to quantitatively compare the genotypes analyzed in this study (Figure 2, B–E): a score of “0” indicated a phenotypically wild-type wing (never observed in the original *wy* mutants); “1” a costal buckle only; “2” a costal buckle along with a distal upturn; and “3” a costal buckle, distal upturn, and shriveled morphology. No other combinations of phenotypes were seen (*e.g.*, distal upturn or shriveled wings without the other two phenotypes). The two wings of a fly were given a collective score because in >99% of flies examined, there was symmetrical penetrance and expressivity, and so both wings would have been given the same score if they had been scored individually. In the rare instance when a phenotypic mismatch was seen between the two wings of a fly, the wings would have always received scores within 1 of each other, and the fly was given the lower of the two scores.

On average, *wy^74i^* had the most severe phenotype, followed by *wy^2^* and *wy^1^* (Table 1A). All three alleles were fully recessive, and no significant sexual dimorphism was observed within any strain. The three mutant strains we obtained have been described by different researchers in publications that were separated by significant spans of time, and we could not find explicit confirmation in the literature that all three alleles map to the same locus (Nachtsheim 1928; Parker 1935; Lindsley and Zimm 1992). Therefore, we crossed all three *wy* strains to each other and examined the wings of heteroallelic F1 females. All three alleles fail to complement one another, supporting the hypothesis that they map to the same locus (Table 1B). The *wy^1^* phenotype became significantly more severe when in a heteroallelic combination with *wy^2^* or *wy^74i^*, and the *wy^2^* phenotype became significantly more severe over *wy^74i^*. These experiments also provided further validation for our numerical scale, since the hierarchical nature of the phenotypes shown in Figure 2, B–E was still seen, even in these mixed genetic backgrounds.

**Table 1:** Wing scores*^a^* of *wy* mutant strains (A), complementation analysis (B), rescue (C), and RNAi (D)

Genotype	n	% With Each Wing Score				Average Wing Score
		0	1	2	3	
A. Mutant strains						
* wy^1^*	31	0.0	83.9	12.9	3.2	1.2*
* wy^2^*	57	0.0	3.5	57.9	38.6	2.3*
* wy^74i^*	42	0.0	0.0	38.1	61.9	2.7*
B. Complementation analysis*^b^*						
* wy^1^*/*wy^2^*	30	0.0	33.3	53.3	13.3	1.8**
* wy^1^*/*wy^74i^*	32	0.0	12.5	84.4	3.1	1.9**
* wy^2^*/*wy^74i^*	33	0.0	0.0	36.4	63.6	2.6**
* wy^1^*/Df	25	0.0	0.0	96.0	4.0	2.0***
* wy^2^*/Df	25	0.0	0.0	96.0	4.0	2.0***
* wy^74i^*/Df	20	0.0	0.0	100.0	0.0	2.0***
C. Rescue*^c^*						
* wy^1^*; UAS*-IP3K2*/+	28	100.0	0.0	0.0	0.0	0.0***
* wy^2^*; UAS-*IP3K2*/+	17	100.0	0.0	0.0	0.0	0.0***
* wy^74i^*; UAS-*IP3K2*/+	39	0.0	100.0	0.0	0.0	1.0***
D. RNAi*^d^*						
* nub*-GAL4/+	47	100.0	0.0	0.0	0.0	0.0
RNAi-*IP3K2*/+	46	100.0	0.0	0.0	0.0	0.0
* nub*-GAL4/+; RNAi-*IP3K2*/+	26	0.0	0.0	88.5	11.5	2.1****

a See first section of *Results* and Figure 2, B–E for a detailed description of the scoring system.

b ”Df” represents the deficiency carried by the Df(1)Exel6245 stock (BL#7718). Details for these crosses are in the *Materials and Methods*.

c One copy of a UAS‐*IP3K2* transgene on chromosome 3 was crossed into *wy* mutant backgrounds (denoted as “*wy*; UAS‐*IP3K2*/+”, with the “+” indicating that the other third chromosome has no rescue construct insertion). The results from only one of our two UAS‐*IP3K2* insertions are reported here, but the other UAS-*IP3K2* insertion yielded identical results.

d For these experiments, *wy^+^* flies with one copy of the *nub*-GAL4 construct only (“*nub*-GAL4/+”, F1 *Sb* males from a cross between RNAi-*IP3K2*/TM3 *Sb* females and *Dcr-2*; *nub*-GAL4 males), one copy of RNAi‐*IP3K2* only (“RNAi‐*IP3K2*/+”, F1 *Sb^+^* males from a cross between RNAi‐*IP3K2*/TM3 *Sb* females and *y^1^ w^1^* males), and both in combination (“*nub*-GAL4/+; RNAi-*IP3K2*/+”, F1 *Sb^+^* males from a cross between RNAi-*IP3K2*/TM3 *Sb* females and *Dcr-2*; *nub*-GAL4 males) were scored and compared. As with footnote c, the “+” denotes a chromosome with no construct insertion. Qualitatively similar results were obtained with female progeny from these same crosses.

* *P* < 10^−14^, Fisher’s exact test comparing each true breeding mutant strain to the other two strains listed in section A of this table.

** *P* < 10^−7^, or in the case of *wy^2^/wy^74i^*, *P* < 0.05, Fisher’s exact test comparing flies with a heteroallelic combination to those only carrying the corresponding milder allele from Section A of this table (*e.g.*, *wy^1^/wy^74i^* compared to *wy^1^*).

*** *P* < 10^–6^, Fisher’s exact test comparing *wy*/Df or *wy*; UAS‐*IP3K2*/+ flies to the corresponding *wy* control from Section A of this table (*e.g.*, *wy^2^*/Df or *wy^2^*; UAS‐*IP3K2*/+ compared to *wy^2^*).

**** *P* < 10^–18^, Fisher’s exact test comparing *nub*-GAL4/+; RNAi-*IP3K2*/+ to its controls *nub*-GAL4/+ and RNAi-*IP3K2*/+ in section D of this table.

We did not observe the lengthened abdomens that were previously reported of *wy^1^* mutants in any of our *wy* mutant strains (Nachtsheim 1928).

### Mapping the *wy* locus

**Figure 3:**
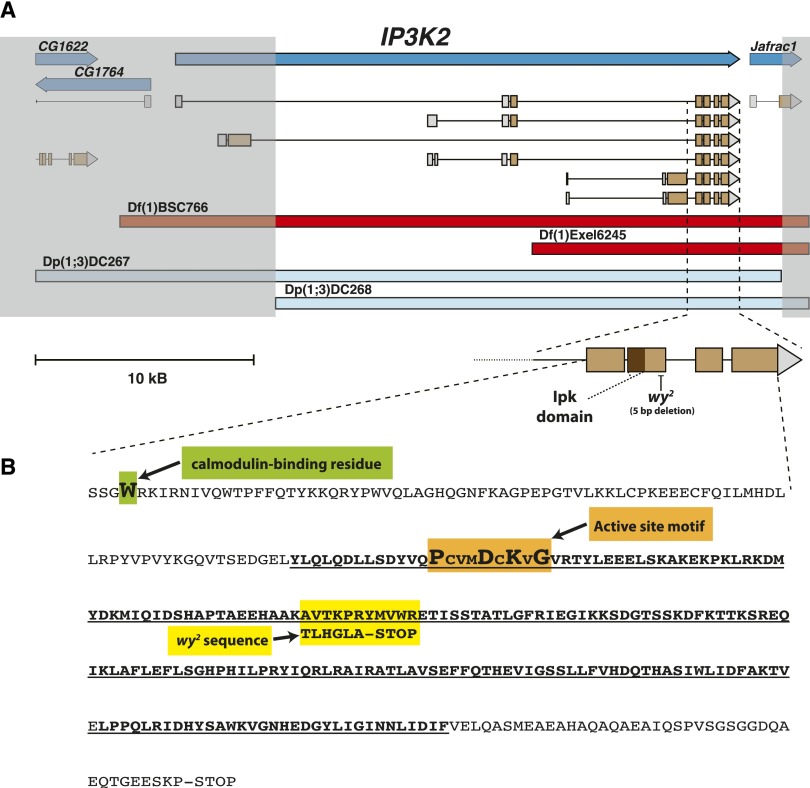
*IP3K2* is the *wavy* gene. (A) Image depicts the 11E9-11 region of the X chromosome, after an image generated using the Flybase GBrowse tool (St Pierre *et al.* 2014). From top, dark blue rectangles represent gene boundaries, with arrowheads indicating directions of transcription. Known transcripts are displayed immediately below gene boundaries, with tan representing coding sequence, gray representing noncoding sequence, lines representing introns, and again arrowheads indicating directions of transcription. Red rectangles indicate sequences deleted in the Df(1)BSC766 and Df(1)Exel6245 stocks. These deletions fail to complement *wy^1^*, *wy^2^*, and *wy^74i^*. On the other hand, the sequences duplicated in the Dp(1;3)DC267 and Dp(1;3)DC268 stocks (light blue rectangles) fully complement all three *wy* alleles. The unshaded region in the middle of the figure highlights the overlap between all four of these deleted and duplicated sequences; *IP3K2* is the only gene that expresses full length transcripts and predicted coding sequences from this shared segment. In further support of *IP3K2* being the *wavy* gene, the *wy^2^* allele contains a 5-bp deletion (GenBank accession number KT732029) just downstream of a conserved inositol polyphosphate kinase (Ipk) domain (further magnified image at bottom right, dark brown segment). Rescue and RNAi experiments also indicated that *IP3K2* is the *wavy* gene (see Table 1, C and D). Bottom left, a scale bar for the top, low-magnification portion of the figure panel. (B) Putative amino acid sequence of the four contiguous coding exons that are shared by all known IP3K2 transcript isoforms [*i.e.*, the enlarged, brown exons featured at the bottom right of (A)]. Green box highlights a tryptophan residue that is necessary for calmodulin binding (Lloyd-Burton *et al.* 2007). Underlined, bold sequence represents a domain that is highly conserved by the Ipk superfamily that includes IP3K enzymes. Orange box highlights a PxxxDxKxG motif, which is a key characteristic of the active site (Lloyd-Burton *et al.* 2007). Yellow box outlines the location and effect of the *wy^2^* mutation: a frameshift that changes the sequence of six amino acids then inserts a premature stop codon.

Standard three-point cross mapping using *forked* (*f*) and various *w^+^*-carrying transposable element insertions within the *wy* region as reference points placed *wy^2^* between PBac{WH}*CG12096^f05782^* (13,159,870) and P{EP}*Tango2^G517^* (13,617,116). This was followed by finer resolution mapping using complementation assays between *wy* alleles, and a series of defined deletions and duplications (Parks *et al.* 2004; Venken *et al.* 2010; Cook *et al.* 2012). The deletions Df(1)BSC766 and Df(1)Exel6245 failed to complement *wy^1^*, *wy^2^*, and *wy^74i^*, and the duplications Dp(1;3)DC267 and Dp(1;3)DC268 fully complemented these same *wy* alleles. The overlapping region between these deletions and duplications implicates *IP3K2* (*IP_3_ 3-kinase 2*) as the *wavy* gene (Figure 3A).

Identifying noncomplementing deletions also provided an opportunity to genetically characterize the available mutant alleles. Over the noncomplementing deletion Df(1)Exel6245, *wy^1^* hemizygotes exhibit a more severe phenotype than homozygotes, while *wy^2^* and *wy^74i^* hemizygotes have a slightly less severe phenotype than homozygotes (Table 1B).

### Sequencing the *wy^2^* allele, rescue, and RNAi

The majority of DNA magnified in Figure 3B was sequenced for *wy^+^* (from the *y^1^ w^1^* strain), *wy^2^*, and *wy^74i^* flies. Consistent with the hypothesis that *IP3K2* is the *wavy* gene, *wy^2^* flies have a 5-bp deletion in the open reading frame of *IP3K2* downstream of the calmodulin-binding site and catalytic domain, presumably causing a frameshift and premature stop codon (Figure 3B; GenBank accession number KT732029). In the case of *wy^74i^*, no mutation was identified in our sequencing of the majority of the region shown in Figure 3B. It is therefore possible that the *wy^74i^* mutation is in an upstream exon, or expression regulatory region.

Flies mutant for *wy^1^* or *wy^2^* were fully rescued, and *wy^74i^* flies were significantly rescued by a single copy of a UAS-*IP3K2* rescue construct, even without a GAL4 driver, probably due to low levels of` leaky expression from the transgene (Table 1C). These results were seen with both of our rescue construct insertions.

To further confirm the identity of the *wavy* gene as *IP3K2*, and to determine if the gene acts within the developing wing itself, we crossed flies carrying the RNAi-*IP3K2* construct to *nub*-GAL4, which expresses GAL4 throughout the prospective wing blade of the wing disc (Brand and Perrimon 1993; Azpiazu and Morata 2000). The *nub*-GAL4-driven RNAi-*IP3K2* recapitulated the *wy* phenotype (Table 1D).

### Temporal requirement for *IP3K2* function

**Figure 4:**
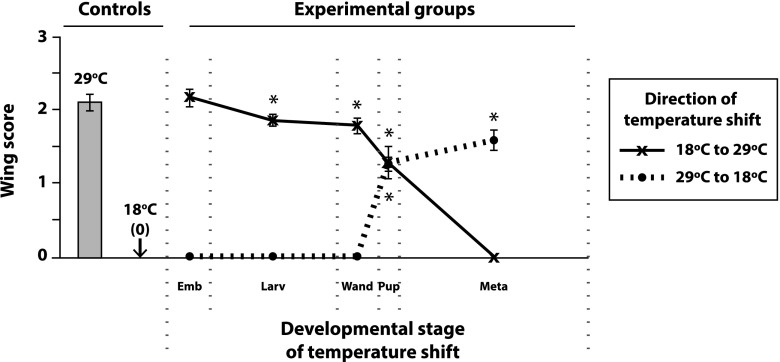
Identifying the critical stage for *IP3K2* function in the developing wing using the GAL4-GAL80^ts^ system and RNAi. *nub*-GAL4 *Tub*-GAL80^ts^ females were mated to RNAi-*IP3K2* males, and their *nub*-GAL4 *Tub*-GAL80^ts^/+; RNAi-*IP3K2*/+ progeny were reared at either 29°C to express the RNAi construct at high levels or at 18°C to minimize its expression. Control groups (left) were reared at either 29°C or 18°C for their entire life cycle and experimental groups (right) were initially reared at one temperature or the other, shifted from 18°C to 29°C (solid line) or from 29°C to 18°C (dotted line) during a specific developmental window, then maintained at the second temperature for the remainder of their life cycles. Developmental windows during which temperature shifts were administered (x-axis) were designated based on published descriptions of the fly life cycle (Bainbridge and Bownes 1981; Bate *et al.* 1993): (1) *Emb*, embryos; (2) *Larv*, first through mid-third instar larvae; (3) *Wand*, late third instar-wandering larvae; (4) *Pup*, white puparium formation-buoyant (P1–P3); and (5) *Meta*, metamorphosis from head eversion to meconium stages (P4–P15). y-axis indicates *wy* phenotype scoring as described in Figure 2, B–E and the *Results* section. Average wing scores are shown with error bars depicting the standard errors of the means. *, *P* < 0.05 for the Fisher’s exact tests comparing the marked experimental group to each of the two controls on the far left. (For all unmarked experimental groups, *P* > 0.05 when tested against one of these two controls, and *P* < < 0.05 when tested against the other control.) Raw data for this experiment are presented in the Table S2.

We next sought to determine the point of development at which *IP3K2* function is required for affecting adult wing morphology using the GAL4-GAL80^ts^ system (Ferris *et al.* 2006; Baena-Lopez *et al.* 2009; Rodriguez *et al.* 2012). Given that the UAS-*IP3K2* construct did not require a GAL4 driver to rescue *wy* mutants, yet a copy of the RNAi-*IP3K2* construct did require a GAL4 driver to phenocopy *wy*, we shifted our focus to RNAi for these experiments so as to control GAL4-driven construct expression with GAL80^ts^ and temperature shifts (see the Figure 4 legend for a detailed description of the experimental design). Control flies that were reared at 29° exhibited the *wy* phenotype, presumably due to dysfunctional GAL80^ts^, and consequent functioning of *nub*-GAL4 to express the RNAi-*IP3K2* construct. Control flies reared at 18° did not phenocopy *wy* at all, presumably because GAL80^ts^ was able to repress GAL4, and because GAL4 is generally less active at this lower temperature (Duffy 2002; Ferris *et al.* 2006; Baena-Lopez *et al.* 2009; Rodriguez *et al.* 2012). Reciprocal shifts from 29° to 18°, and from 18° to 29°, at various points during the life cycle revealed a dramatic reduction in the frequency of the *wy* phenotype if flies had experienced their Stages P1–P3 (white puparium-buoyant, see Bainbridge and Bownes 1981) at 18° (low RNAi-*IP3K2* expression) as opposed to 29° (high RNAi-*IP3K2* expression). This suggests that *IP3K2* function is required in the prospective wing blade for wing development during early pupal life.

### Genetic interaction assays between *wy* and other IP_3_ signaling loci

Given that *IP3K2* encodes an IP_3_ 3-kinase, other components of IP_3_ signaling may interact with *IP3K2* to affect wing development. To investigate this hypothesis, we tested whether mutations in several different IP_3_ pathway loci dominantly modify the *wy* phenotype, reasoning that such a sensitive interaction would indicate a strong functional relationship. The *wy^2^* allele was used for the primary screen because of its intermediate phenotype, and therefore presumed versatility in detecting both genetic enhancers and suppressors. *IP3K1*, *Ipk2*, *Cam*, and *IP3R* were selected as candidate interactors because the proteins encoded by these loci have strong biochemical associations with IP3K2: IP3K1 and Ipk2 also use IP_3_ as a substrate, Cam binds to and regulates IP3K2, and IP3R binds IP_3_ (Banerjee *et al.* 2004; Seeds *et al.* 2004; Lloyd-Burton *et al.* 2007).

In a *wy^+^* background, the *IP3K1^KG02192^*, Df-*Ipk2*, *Cam^n339^*, *Cam^7^*, *IP3R^90B.0^*, and *IP3R^ug3^* alleles were all homozygous lethal mutations, and had no discernible effect on wing structure in the heterozygous condition. *IP3R^sa54^*/*IP3R^ug3^* mutants had been reported to be a viable heteroallelic combination that exhibited mild crumpling at the margins of their wings, but we were unable to obtain the *IP3R^sa54^* allele to reproduce these results (Banerjee *et al.* 2004). In our genetic modifier screen, *IP3K1^KG02192^*, Df-*Ipk2*, *Cam^n339^*, and *Cam^7^* did not dominantly modify *wy^2^* wing scores (Table S3), but *IP3R^90B.0^* and *IP3R^ug3^* did (Table 2), strongly suppressing the *wy^2^* phenotype relative to controls (for all modifier tests, controls were *wy*; ^+^/ ^+^ siblings from the same cross–cross schemes described in the footnotes of Table S3 and Table 2). Further tests showed that the phenotypes of *wy^74i^*, and especially *wy^1^* flies, were also dominantly suppressed by both *IP3R* alleles—in fact, the wings of virtually all *wy^1^*; *IP3R^ug3^/*+ and *wy^1^*; *IP3R^90B.0^*/+ flies were phenotypically wild type (Table 2). In summary, all three mutant alleles of *wy* were dominantly suppressed by both mutant alleles of *IP3R*.

**Table 2:** Testing for dominant modification of the *wy* phenotype by loss of function in the IP_3_ receptor gene *IP3R**^a^*^,^*^b^*

Genotype	*n*	% With Each Wing Score				Average Wing Score
		0	1	2	3	
*wy^2^*; *IP3R^ug3^*/+	25	28.0	72.0	0.0	0.0	0.7*
*wy^2^*; +/+	25	16.0	40.0	44.0	0.0	1.3
*wy^2^*; *IP3R^90B.0^*/+	25	8.0	40.0	52.0	0.0	1.4*
*wy^2^*; +/+	25	4.0	4.0	68.0	24.0	2.1
*wy^1^*; *IP3R^ug3^*/+	25	100.0	0.0	0.0	0.0	0.00*
*wy^1^*; +/+	15	33.3	60.0	6.7	0.0	0.70
*wy^1^*; *IP3R^90B.0^*/+	25	96.0	4.0	0.0	0.0	0.04*
*wy^1^*; +/+	25	40.0	44.0	12.0	4.0	0.80
*wy^74i^*; *IP3R^ug3^*/+	22	0.0	0.0	90.9	9.1	2.1**
*wy^74i^*; +/+	25	0.0	0.0	64.0	36.0	2.4
*wy^74i^*; *IP3R^90B.0^*/+	25	0.0	0.0	100.0	0.0	2.0*
*wy^74i^*; +/+	17	0.0	0.0	0.0	100.0	3.0

The wings of *wy*/Y; [*IP3R* mutant allele]/+, F1 males (experimental group, upper row within each pair) were compared to those of their *wy*/Y; +/TM6B *Tb^1^*, *IP3R^+^* F1 male siblings (control group, lower row within each pair)note that in the table, TM6B *Tb^1^*, *IP3R^+^* is shortened to a second + for simplicity). These comparisons tested whether mutations in *IP3R* can dominantly modify *wy* while controlling for the genetic background of the other chromosomes. While the change in genetic background or even the TM6B balancer may have had some effect on the distribution of *wy* wing scores, mutations in *IP3R* had a far more pronounced effect, lowering scores significantly relative to their sibling controls and event more dramatically relative to the original strains (compare control and experimental values in this table to Table 1A), the hierarchical nature of the phenotypes was once again preserved and no novel phenotypes were seen.

a See first section of *Results* and Figure 2, B–E for a detailed description of the scoring system.

b Each pair of rows compares F1 male siblings from the following cross: (*wy*/*wy*; +/+ females) X (*wy^+^*/Y; [*IP3R* mutant allele]/TM6B *Tb^1^*, *IP3R^+^* males)

* *P* < 10^–4^, Fisher’s exact test *vs.* sibling controls in the row immediately below the marked row.

** *P* < 0.05, Fisher’s exact test *vs.* sibling controls in the row immediately below the marked row.

## Discussion

### *wavy* maps to *IP_3_ 3-kinase 2*

In this study, we present strong evidence that mutations in *wavy* (*wy*), the first of which was described nearly 90 years ago (Nachtsheim 1928), are alleles of *IP3K2* (*IP_3_ 3-kinase 2*). The three-point recombination mapping, along with complementation analyses using molecularly defined deletions and duplications, mapped *wy* down to the *IP3K2* gene (Figure 3A). Sequencing of the *IP3K2* gene of *wy^2^* flies revealed a 5-bp deletion in its open reading frame, putatively causing a frameshift mutation (Figure 3B; GenBank accession number KT732029), and, although mutant sequences have not yet been identified for the other two alleles, *wy^2^* fails to complement *wy^1^* and *wy^74i^* (Table 1B), suggesting that they are alleles of the same locus. A UAS-*IP3K2* construct rescues all three *wy* alleles (Table 1C), and RNAi of *IP3K2* using the wing disc-specific driver *nub*-GAL4 phenocopies *wy* (Table 1D).

### How the available *wy* alleles might affect IP3K2 function

*wy^1^*/Df flies have a more severe phenotype than *wy^1^*/*wy^1^* flies, suggesting that *wy^1^* is a hypomorphic allele. On the other hand, *wy^2^* and *wy^74i^* become less severe in the hemizygous condition, yet both are fully recessive (Table 1B). Hence, *wy^2^* and *wy^74i^* do not neatly fall into any classic mutant category (Muller 1932; Wilkie 1994). However, the UAS-*IP3K2* construct fully rescues *wy^2^* and significantly alleviates the *wy^74i^* phenotype, and RNAi-*IP3K2* expression in the wing disc causes a phenotype that resembles those of both *wy^2^* and *wy^74i^* (Table 1, C and D). Finally, both alleles are fully complemented by the duplications shown in Figure 3A. Taken together, these data suggest that *wy^2^* and *wy^74i^* are both strong loss-of-function alleles, and that their wing scores were somewhat reduced by the genetic background of the deficiency line. A molecular null allele of *IP3K2* was recently generated (Nelson *et al.* 2014). Although no mention was made of a wing-related phenotype, the reporting manuscript was wholly focused on the function of *IP3K2* in the salivary glands. Analysis of how this null allele affects wing development would provide further insight into the nature of the *wy^2^* and *wy^74i^* alleles and, of course, be necessary to understand the consequences of completely removing gene function.

The frameshift mutation we found in *wy^2^* lies well downstream of the regions encoding a calmodulin-binding domain and the active site (Figure 3B). Therefore, *wy^2^* may have some IP3K2 activity in spite of its strong phenotype. Consistent with this hypothesis, *wy^2^* is fully rescued by UAS-*IP3K2* without any GAL4 driver, while UAS-*IP3K2* only partially rescues the more severe *wy^74i^* allele (Table 1C). Even if *wy^2^* had some residual function, the protein encoded by *wy^2^* would be truncated by a premature stop codon, and so its conformation, interactions with regulating proteins such as calmodulin (Lloyd-Burton *et al.* 2007), and/or stability may be significantly affected. Enzymatic assays of the altered IP3K2 enzyme encoded by *wy^2^* may provide further insight into how this allele affects enzyme activity, stability, and regulation. Similar studies could be done with proteins encoded by the *wy^1^* and *wy^74i^* alleles once they have been molecularly defined.

### *IP3K2* function is required in the developing wing blade during early pupal life

Controlled expression of the RNAi-*IP3K2* construct using *nub*-GAL4, *Tub*-GAL80^ts^, and temperature shifts during specific developmental windows revealed a requirement for *IP3K2* function in the wing disc during pupal stages P1–P3 (Figure 4 and Table S2). This developmental window may provide clues as to the cellular process that *IP3K2* is involved in. As described in the *Introduction* and in Figure 1, IP_3_ signaling can regulate IP3R-mediated Ca^2+^ release from stores in the ER, and, elsewhere in the literature, there is evidence that intracellular calcium signaling is involved in the development of the pupal wing of insects. Cytoplasmic calcium waves have been documented in the pupal wings of the butterfly *Junonia orithya*, and these waves are halted by pharmacological inhibition of ER Ca^2+^-ATPase, an enzyme responsible for initially sequestering Ca^2+^ in the ER before release occurs. Data suggest that these calcium waves are involved in wing eyespot development in *Junonia orithya* (Ohno and Otaki 2015). Calcium waves have also been induced in *Drosophila* larval wing discs in response to laser-induced wounding (Narciso *et al.* 2015).

The *Drosophila* Cam protein binds and regulates the activity of IP3K2 in a Ca^2+^-dependent fashion (Figure 3B; Lloyd-Burton *et al.* 2007). Many loss of function alleles in *Cam* are lethal before adulthood, but some viable alleles result in ectopic wing veins (Nelson *et al.* 1997). While we did not observe this ectopic vein phenotype with loss of *IP3K2* function or with our genetic interaction experiments, Cam has a broad spectrum of functions, and so the ectopic vein phenotype may be due to a process unrelated to IP_3_ signaling. Relevant here, however, is that the wing vein positions are refined during the P1–P3 stages, the stages at which we found *IP3K2* function is required in the wing (Blair 2007; Figure 4 and Table S2). In addition, it is at least known that *Cam* mRNA is expressed at very high levels in wing discs that were cultured shortly before the P1 stage (Cherbas *et al.* 2011). Therefore Cam, a Ca^2+^-dependent regulator of IP3K2 activity, may be active in the wing disc during the same developmental window in which *IP3K2* function is required (Figure 4). Although our dominant modifier screen did not detect an interaction between *wy* and *Cam* (Table S3), a single copy of a *Cam* mutation may not have reduced function enough to see an effect.

Similarly, we did not detect dominant modification of *wy* by mutations in *IP3K1* or *Ipk2* (Table S3). Both *IP3K1* and *Ipk2* encode enzymes that are specific for the IP3K2 substrate (IP_3_) and, similar to *Cam*, both genes are expressed at moderate-to-high levels in the cultured wing discs of wandering larvae (Seeds *et al.* 2004; Cherbas *et al.* 2011). Therefore, IP3K2, IP3K1, and Ipk2 might compete for the same IP_3_ pool in the wing discs, and/or exhibit redundant functions. Importantly, however, our experiments indicate a requirement for *IP3K2* function at the P1–P3 stages—not during but shortly after the wandering phase (Figure 4), and, to our knowledge, detailed expression patterns are not available for *IP3K2*, *Cam*, *IP3K1*, *Ipk2*, or *IP3R* in wing discs during these developmental stages. To resolve this ambiguity, future experiments should characterize expression of, and more extensively test interactions between, these IP_3_ signaling pathway genes, focusing on the early pupal wing disc, and using stronger losses of gene functions than were present in our dominant modifier screen.

While we have determined a spatiotemporal requirement for *IP3K2* function, and an interacting locus (*IP3R*), our data do not identify a cellular mechanism underlying the *wy* phenotype. The developmental events that normally occur in the wing disc during the P1–P3 stages may provide clues into this aspect of *IP3K2* function. For example, wing bristle precursors at the anterior margin of the wing proliferate during early pupal life, while cells of the prospective wing blade are mitotically quiescent until shortly after P3 (Milán *et al.* 1996b). Interestingly, the anterior margin is the general region of the wing that most consistently exhibits a phenotype in *wy* flies (*i.e.*, the “costal buckles” shown in Figure 2 A and C–E). IP_3_ signaling is involved in the cell proliferation of multiple systems, and, in *Drosophila*, *IP3R* has been shown to be required for the cytokinesis of spermatocytes (Wong *et al.* 2005; Berridge 2009; Leanza *et al.* 2013; Nohara *et al.* 2015). Therefore, it is conceivable that mutations in *wy* disrupt cell cycle regulation in the pupal wing.

Another possible function for *IP3K2* in the wing comes from reports of its function in another *Drosophila* tissue. The micro-RNA *miR-14* induces autophagy of the salivary glands during early pupal life by targeting *IP3K2* (Nelson *et al.* 2014). The consequent downregulation of *IP3K2* is thought to increase the amount of IP_3_ available to *IP3R*, *IP3R* is activated as a result, and autophagy is induced, at least in part by Ca^2+^ release from the ER. This same study suggested that *Atg6*, an autophagy-inducing gene that encodes a component of the Vps34 phosphatidylinositol 3-kinase (PI3K) complex III, acts in the same pathway as *miR-14*. In another study, *Atg6* was shown to be required for autophagy in the pupal wing of *Drosophila* (Lőrincz *et al.* 2014). These findings suggest the intriguing possibility that *IP3K2* and *IP3R* regulate autophagy in the developing wing, perhaps by interactions with the *Atg6*/*miR-14* module.

### Modeling the interactions between *wavy* and *IP3R*

In past studies, mild wing crumpling in *IP3R^sa54^*/*IP3R^ug3^* mutants hinted at the involvement of the IP_3_ signaling network in wing development, but further analysis was presumably hindered because other *IP3R* allele combinations were either lethal or had normal wing morphology (Banerjee *et al.* 2004). *wy* provides an alternative entry point to *IP3R*, and has useful qualities for investigating how IP_3_ signaling affects wing morphology: (1) flies with strong loss of *IP3K2* function have good viability; and (2) the *wy* phenotype has several discrete features to it—costal buckling, upward curling, and overall crumpling—that are easily scored and consistently appear in a hierarchical pattern (Figure 2, B–E and Table 1). This makes the *wy* phenotype an efficient, precise indicator of the levels of *IP3K2* gene and overall IP_3_ pathway function, and therefore a sensitive gauge for identifying genetic interactors.

**Figure 5:**
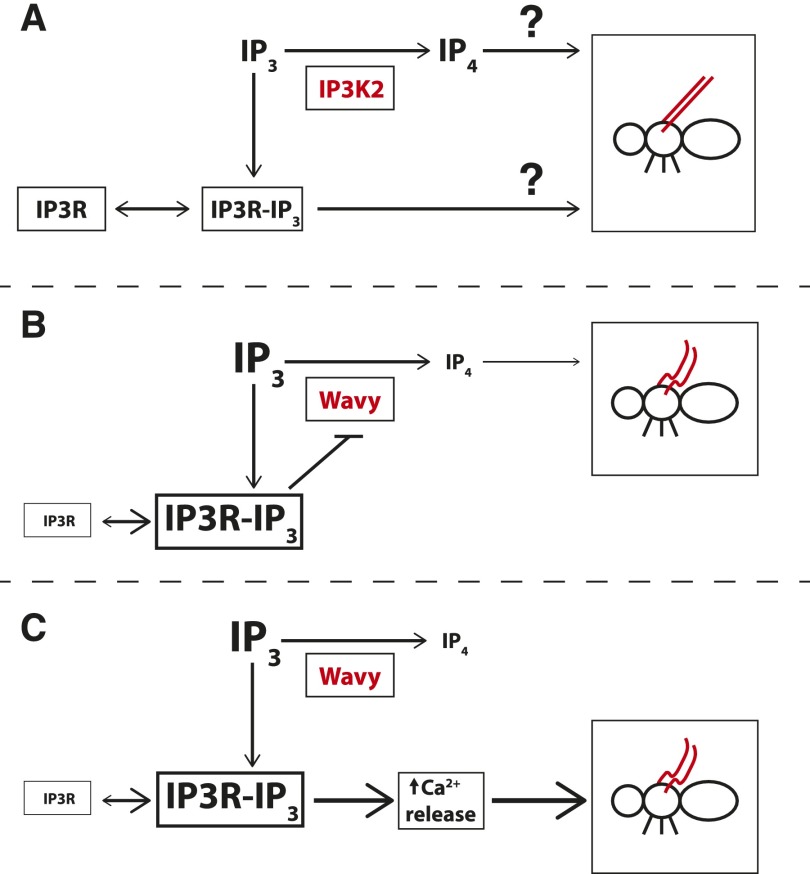
Potential models for how *IP3K2* affects wing morphology and how mutations in *IP3R* dominantly suppress the *wy* phenotype. (A) The strong genetic interaction between *wy* and *IP3R*, and what is known about the biochemical functions of their encoded proteins, suggest that wing morphology as assessed in this study may be affected by IP_4_ signaling, IP_4_-independent IP3R signaling, or an integration of both signals. Question marks indicate uncertainty about the relative importance of these two signals. By extension, the *wy* phenotype may be caused by (B) reduced IP_4_ levels, and/or (C) excessive IP3R activation that triggers IP_4_-independent signals (*e.g.*, increased Ca^2+^ release from the ER). (B) If the *wy* phenotype is caused solely by reduced IP_4_ levels, then IP3R would be expected to further inhibit accumulation of IP_4_ by inhibiting residual activity of mutant IP3K2 enzyme (“Wavy”), for example, by usurping the IP_3_ substrate. A mutant copy of *IP3R* would be expected to make more IP_3_ available to IP3K2, increasing IP_4_ formation and suppressing the *wy* phenotype. Increased levels of IP_3_ are shown here due to loss of *IP3K2* function. However, this model would hold whether or not IP_3_ actually accumulates in the wing discs of *wy* mutants, because in either case, loss of IP3R function could increase the amount of substrate available to the mutant IP3K2 enzyme. (C) Model if increased IP3R signaling triggers downstream, IP_4_-independent events to cause the *wy* phenotype. Here, IP_3_ is assumed to accumulate due to loss of *IP3K2* function, as suggested by studies in *Drosophila* S2 cells (Seeds *et al.* 2004); this accumulation of IP_3_ would be expected to hyperactivate IP3R, increasing calcium release from the ER. A partial loss of *IP3R* function would reduce this excessive IP3R signaling, suppressing the *wy* phenotype. (B) and (C) represent extreme models that exclude one factor or the other, but a hybrid model is also possible where both IP_4_ signaling and IP_4_-independent IP3R signaling play significant roles in wing development.

Our results, along with the biochemical relationship between IP3K2 and IP3R, suggest that IP_4_ levels and/or IP_4_-independent IP3R signaling affect wing development (Figure 5A). Previous studies of *Drosophila* S2 cell cultures have found that loss of *IP3K2* function can contribute to expansion of the IP_3_ pool. This suggests that IP_3_ is being steadily produced from *IP3K2*-independent sources (*e.g.*, phospholipase C), and so, in control cells, the forward reaction for IP3K2 (IP_3_→IP_4_) is predominant (Seeds *et al.* 2004). If we assume that the forward reaction is predominant in the pupal wing tissue as well (which would require direct confirmation in future studies), then loss of *wy* function would be expected to expand the IP_3_ pool, and decrease levels of IP_4_. If this were the case, the *wy* phenotype may be due to insufficient levels of IP_4_ (Figure 5B), and/or an excess of IP_3_ that hyperactivates IP3R (Figure 5C). In both cases, a partial loss of *IP3R* function could potentially alleviate the *wy* mutant phenotype. Both models assume that IP3K2 catalytic activity is required to affect wing morphology. Although we have not tested this model directly, the strong genetic interaction between *wy* and *IP3R* (Table 2), and the biochemical relationship between their encoded proteins, support this assumption. In addition, IP3Ks are typically cytoplasmic (Xia and Yang 2005), and IP3K2 has been shown to localize to the cytoplasm when expressed in HeLa cells (Lloyd-Burton *et al.* 2007). Therefore, IP3K2 protein is likely to be expressed in the proper subcellular compartment in order to have the hypothesized interaction with ER-bound IP3R (*i.e.*, drawing from the same pool of IP_3_).

In summary, this study maps a classic mutant phenotype to a single gene and helps establish *Drosophila* wing development as an effective system to study IP_3_ signaling. Future experiments should investigate possible cellular mechanisms underlying the *wy* phenotype (*e.g.*, the potential roles of Ca^2+^ release from the ER and possible effects of *wy* on cell proliferation and autophagy), as well as continue to test and refine models of how IP3K2 interacts with other components of the IP_3_ signaling network to build a wing.

## Supplementary Material

Supporting Information

## References

[bib1] Agrawal N. Padmanabhan N. Hasan G., 2009 Inositol 1,4,5-trisphosphate receptor function in *Drosophila* insulin producing cells. PLoS One 4: e6652. 10.1371/journal.pone.0006652 PMC2721413 19680544

[bib2] Agrawal N. Venkiteswaran G. Sadaf S. Padmanabhan N. Banerjee S., 2010 Inositol 1,4,5-trisphosphate receptor and dSTIM function in *Drosophila* insulin-producing neurons regulates systemic intracellular calcium homeostasis and flight. J. Neurosci. 30: 1301–1313. 20107057 10.1523/JNEUROSCI.3668-09.2010 PMC6633787

[bib3] Ambudkar I. S., 2014 Ca2+ signaling and regulation of fluid secretion in salivary gland acinar cells. Cell Calcium 55: 297–305. 24646566 10.1016/j.ceca.2014.02.009 PMC4059182

[bib4] Azpiazu N. Morata G., 2000 Function and regulation of homothorax in the wing imaginal disc of *Drosophila*. Development 127: 2685–2693. 10821766 10.1242/dev.127.12.2685

[bib5] Baena-Lopez L. A. Franch-Marro X. Vincent J. P., 2009 Wingless promotes proliferative growth in a gradient-independent manner. Sci. Signal. 2: Ra60. 10.1126/scisignal.2000360 PMC3000546 19809090

[bib6] Bainbridge S. P. Bownes M., 1981 Staging the metamorphosis of *Drosophila melanogaster*. J. Embryol. Exp. Morphol. 66: 57–80. 6802923

[bib7] Banerjee S. Lee J. Venkatesh K. Wu C. F. Hasan G., 2004 Loss of flight and associated neuronal rhythmicity in inositol 1,4,5-trisphosphate receptor mutants of *Drosophila*. J. Neurosci. 24: 7869–7878. 15356199 10.1523/JNEUROSCI.0656-04.2004 PMC1289272

[bib8] Banerjee S. Joshi R. Venkiteswaran G. Agrawal N. Srikanth S., 2006 Compensation of inositol 1,4,5-trisphosphate receptor function by altering sarco-endoplasmic reticulum calcium ATPase activity in the *Drosophila* flight circuit. J. Neurosci. 26: 8278–8288. 16899722 10.1523/JNEUROSCI.1231-06.2006 PMC6673814

[bib9] Bate M. Martinez Arias A. Hartenstein V., 1993 The Development of Drosophila melanogaster, Cold Spring Harbor Laboratory Press, Plainview, NY

[bib10] Bellen H. J. Levis R. W. Liao G. C. He Y. C. Carlson J. W., 2004 The BDGP gene disruption project: single transposon insertions associated with 40% of *Drosophila* genes. Genetics 167: 761–781. 15238527 10.1534/genetics.104.026427 PMC1470905

[bib11] Berridge M. J., 2009 Inositol trisphosphate and calcium signalling mechanisms. Biochim. Biophys. Acta. 1793: 933–940. 19010359 10.1016/j.bbamcr.2008.10.005

[bib12] Biehs B. Sturtevant M. A. Bier E., 1998 Boundaries in the *Drosophila* wing imaginal disc organize vein-specific genetic programs. Development 125: 4245–4257. 9753679 10.1242/dev.125.21.4245

[bib13] Blair S. S., 2007 Wing vein Patterning in *Drosophila* and the analysis of intercellular signaling. Annu. Rev. Cell Dev. Biol. 23: 293–319. 17506700 10.1146/annurev.cellbio.23.090506.123606

[bib14] Brand A. H. Perrimon N., 1993 Targeted gene-expression as a means of altering cell fates and generating dominant phenotypes. Development 118: 401–415. 8223268 10.1242/dev.118.2.401

[bib15] Byrum J. Jordan S. Safrany S. T. Rodgers W., 2004 Visualization of inositol phosphate-dependent mobility of Ku: depletion of the DNA-PK cofactor InsP(6) inhibits Ku mobility. Nucleic Acids Res. 32: 2776–2784. 15150344 10.1093/nar/gkh592 PMC419599

[bib16] Casso D. J. Biehs B. Kornberg T. B., 2011 A novel interaction between hedgehog and notch promotes proliferation at the anterior-posterior organizer of the *Drosophila* wing. Genetics 187: 485–499. 21098717 10.1534/genetics.110.125138 PMC3030491

[bib17] Cavodeassi F. Rodriguez I. Modolell J., 2002 Dpp signalling is a key effector of the wing-body wall subdivision of the *Drosophila* mesothorax. Development 129: 3815–3823. 12135920 10.1242/dev.129.16.3815

[bib88] Cherbas L. Willingham A. Zhang D. Yang L. Zou Y., 2011 The transcriptional diversity of 25 *Drosophila* cell lines. Genome Res. 21: 301–14. 21177962 10.1101/gr.112961.110 PMC3032933

[bib18] Classen A. K. Anderson K. I. Marois E. Eaton S., 2005 Hexagonal packing of *Drosophila* wing epithelial cells by the planar cell polarity pathway. Dev. Cell 9: 805–817. 16326392 10.1016/j.devcel.2005.10.016

[bib19] Classen A. K. Aigouy B. Giangrande A. Eaton S., 2008 Imaging *drosophila* pupal wing morphogenesis, Methods in molecular biology: Drosophila: methods and protocols, edited by Dahmann C. Humana Press Inc., Totowa, NJ. 10.1007/978-1-59745-583-1_16 18641953

[bib90] Cohen B. Simcox A. A. Cohen S. M., 1993 Allocation of the thoracic imaginal primordia in the *Drosophila* embryo. Development 117: 597–608. 8330530 10.1242/dev.117.2.597

[bib20] Cook R. K. Christensen S. J. Deal J. A. Coburn R. A. Deal M. E., 2012 The generation of chromosomal deletions to provide extensive coverage and subdivision of the *Drosophila melanogaster* genome. Genome Biol. 13: R21. 22445104 10.1186/gb-2012-13-3-r21 PMC3439972

[bib21] Crozatier M. Glise B. Vincent A., 2002 Connecting Hh, Dpp and EGF signalling in patterning of the *Drosophila* wing; the pivotal role of collier/knot in the AP organiser. Development 129: 4261–4269. 12183378 10.1242/dev.129.18.4261

[bib22] Crozatier M. Glise B. Vincent A., 2004 Patterns in evolution: veins of the *Drosophila* wing. Trends Genet. 20: 498–505. 15363904 10.1016/j.tig.2004.07.013

[bib23] Daniels R. W. Rossano A. J. Macleod G. T. Ganetzky B., 2014 Expression of multiple transgenes from a single construct using viral 2A peptides in *Drosophila*. PLoS One 9: e100637. 10.1371/journal.pone.0100637 PMC4063965 24945148

[bib24] Dietzl G. Chen D. Schnorrer F. Su K. C. Barinova Y., 2007 A genome-wide transgenic RNAi library for conditional gene inactivation in *Drosophila*. Nature 448: 151–156. 17625558 10.1038/nature05954

[bib25] Donie F. Hulser E. Reiser G., 1990 High-affinity inositol 1,3,4,5-tetrakisphosphate receptor from cerebellum—solubilization, partial-purification and characterization. FEBS Lett. 268: 194–198. 2166682 10.1016/0014-5793(90)81006-a

[bib26] Du J. Zhang J. Z. Su Y. Liu M. Ospina J. K., 2011 In vivo RNAi screen reveals neddylation genes as novel regulators of hedgehog signaling. PLoS One 6: e24168. 10.1371/journal.pone.0024168 PMC3169580 21931660

[bib27] Duffy J. B., 2002 GAL4 system in *Drosophila*: a fly geneticist’s Swiss army knife. Genesis 34: 1–15. 12324939 10.1002/gene.10150

[bib28] Dui W. Lu W. Ma J. Jiao R. J., 2012 A systematic phenotypic screen of F-box genes through a tissue-specific RNAi-based approach in *Drosophila*. J. Genet. Genomics 39: 397–413. 22884096 10.1016/j.jgg.2012.05.009

[bib29] Fain J. N., 2013 *Lipid metabolism in signaling systems*, Academic Press, Waltham.

[bib30] Ferris J. Ge H. Liu L. Z. Roman G., 2006 G(o) signaling is required for *Drosophila* associative learning. Nat. Neurosci. 9: 1036–1040. 16845387 10.1038/nn1738

[bib31] Fukuda M. Mikoshiba K., 1997 The function of inositol high polyphosphate binding proteins. BioEssays 19: 593–603. 9230692 10.1002/bies.950190710

[bib32] Hanakahi L. A. Bartlet-Jones M. Chappell C. Pappin D. West S. C., 2000 Binding of inositol phosphate to DNA-PK and stimulation of double-strand break repair. Cell 102: 721–729. 11030616 10.1016/s0092-8674(00)00061-1

[bib33] Hartl T. A. Scott M. P., 2014 Wing tips: the wing disc as a platform for studying Hedgehog signaling. Methods 68: 199–206. 24556557 10.1016/j.ymeth.2014.02.002

[bib34] Heiman R. G. Atkinson R. C. Andruss B. F. Bolduc C. Kovalik G. E., 1996 Spontaneous avoidance behavior in *Drosophila* null for calmodulin expression. Proc. Natl. Acad. Sci. USA 93: 2420–2425. 8637889 10.1073/pnas.93.6.2420 PMC39812

[bib35] Hoskins R. A. Landolin J. M. Brown J. B. Sandler J. E. Takahashi H., 2011 Genome-wide analysis of promoter architecture in *Drosophila melanogaster*. Genome Res. 21: 182–192. 21177961 10.1101/gr.112466.110 PMC3032922

[bib36] Humbert J. P. Matter N. Artault J. C. Koppler P. Malviya A. N., 1996 Inositol 1,4,5-trisphosphate receptor is located to the inner nuclear membrane vindicating regulation of nuclear calcium signaling by inositol 1,4,5-trisphosphate. Discrete distribution of inositol phosphate receptors to inner and outer nuclear membranes. J. Biol. Chem. 271: 478–485. 8550605 10.1074/jbc.271.1.478

[bib37] Ivanova H. Vervliet T. Missiaen L. Parys J. B. De Smedt H., 2014 Inositol 1,4,5-trisphosphate receptor-isoform diversity in cell death and survival. Biochim. Biophys. Acta 1843: 2164–2183. 24642269 10.1016/j.bbamcr.2014.03.007

[bib38] Joshi R. Venkatesh K. Srinivas R. Nair S. Hasan G., 2004 Genetic dissection of itpr gene function reveals a vital requirement in aminergic cells of *Drosophila* larvae. Genetics 166: 225–236. 15020420 10.1534/genetics.166.1.225 PMC1470716

[bib39] Kaneuchi T. Sartain C. V. Takeo S. Horner V. L. Buehner N. A., 2015 Calcium waves occur as *Drosophila* oocytes activate. Proc. Natl. Acad. Sci. USA 112: 791–796. 25564670 10.1073/pnas.1420589112 PMC4311822

[bib40] Kiger J. A. Natzle J. E. Kimbrell D. A. Paddy M. R. Kleinhesselink K., 2007 Tissue remodeling during maturation of the *Drosophila* wing. Dev. Biol. 301: 178–191. 16962574 10.1016/j.ydbio.2006.08.011 PMC1828914

[bib41] Klein T., 2001 Wing disc development in the fly: the early stages. Curr. Opin. Genet. Dev. 11: 470–475. 11448635 10.1016/s0959-437x(00)00219-7

[bib42] Kwon, M. H., H. Callaway, J. Zhong and B. Yedvobnick, 2013 A targeted genetic modifier screen links the SWI2/SNF2 protein domino to growth and autophagy genes in *Drosophila melanogaster*. G3 (Bethesda) 3: 815–825. 10.1534/g3.112.005496 PMC3656729 23550128

[bib43] Lahr E. C. Dean D. Ewer J., 2012 Genetic analysis of ecdysis behavior in *Drosophila* reveals partially overlapping functions of two unrelated neuropeptides. J. Neurosci. 32: 6819–6829. 22593051 10.1523/JNEUROSCI.5301-11.2012 PMC6622212

[bib44] Leanza L. Biasutto L. Manago A. Gulbins E. Zoratti M., 2013 Intracellular ion channels and cancer. Front. Physiol. 4: 227. 24027528 10.3389/fphys.2013.00227 PMC3759743

[bib45] Lindsley D. L. Zimm G. G., 1992 The genome of *Drosophila melanogaster*. Science 257: 1421–1422.17738283

[bib46] Link N. Chen P. Lu W. J. Pogue K. Chuong A., 2007 A collective form of cell death requires homeodomain interacting protein kinase. J. Cell Biol. 178: 567–574. 17682052 10.1083/jcb.200702125 PMC2064464

[bib47] Lloyd-Burton S. M. Yu J. C. H. Irvine R. F. Schell M. J., 2007 Regulation of inositol 1,4,5-trisphosphate 3-kinases by calcium and localization in cells. J. Biol. Chem. 282: 9526–9535. 17284449 10.1074/jbc.M610253200

[bib48] Lőrincz, P., Z. Lakatos, T. Maruzs, Z. Szatmaari, V. Kis *et al.*, 2014 Atg6/UVRAG/Vps34-containing lipid kinase complex is required for receptor downregulation through endolysosomal degradation and epithelial polarity during *Drosophila* wing development. Biomed Res. Int. 2014: 851349 10.1155/2014/851349 PMC4074780 25006588

[bib49] Ma Y. M. Lieber M. R., 2002 Binding of inositol hexakisphosphate (IP6) to Ku but not to DNA-PKcs. J. Biol. Chem. 277: 10756–10759. 11821378 10.1074/jbc.C200030200

[bib50] Macbeth M. R. Schubert H. L. VanDemark A. P. Lingam A. T. Hill C. P., 2005 Inositol hexakisphosphate is bound in the ADAR2 core and required for RNA editing. Science 309: 1534–1539. 16141067 10.1126/science.1113150 PMC1850959

[bib51] Malviya A. N. Klein C., 2006 Mechanism regulating nuclear calcium signaling. Can. J. Physiol. Pharmacol. 84: 403–422. 16902586 10.1139/y05-130

[bib52] Milán, M., and S. Campuzano, and A. GarciaBellido, 1996a Cell cycling and patterned cell proliferation in the *Drosophila* wing during metamorphosis. Proc. Natl. Acad. Sci. USA 93: 11687–11692. 10.1073/pnas.93.21.11687 PMC38119 8876197

[bib53] Milán, M., and S. Campuzano, and A. GarciaBellido, 1996b Cell cycling and patterned cell proliferation in the wing primordium of *Drosophila*. Proc. Natl. Acad. Sci. USA 93: 640–645. 10.1073/pnas.93.2.640 PMC40104 8570608

[bib54] Muller H. J., 1932 Further studies on the nature and causes of gene mutations, pp. 213–255 in *6th International Congress of Genetics*. 1: 213

[bib55] Nachtsheim H., 1928 Beitrag zur Topographie des X–Chromosoms von *Drosophila melanogaster*. Z. Indukt. Abstamm 48: 245–258.

[bib56] Narciso, C., Q. Wu, P. Brodskiy, G. Garston, R. Baker, *et al.* 2015 Patterning of wound-induced intracellular Ca^2+^ flashes in a developing epithelium. Phys. Biol. 12: 056005. 10.1088/1478-3975/12/5/056005 PMC4605135 26331891

[bib57] Natzle J. E. Kiger J. A. Green M. M., 2008 Bursicon signaling mutations separate the epithelial-mesenchymal transition from programmed cell death during *Drosophila melanogaster* wing maturation. Genetics 180: 885–893. 18780731 10.1534/genetics.108.092908 PMC2567388

[bib58] Nelson H. B. Heiman R. G. Bolduc C. Kovalick G. E. Whitley P., 1997 Calmodulin point mutations affect *Drosophila* development and behavior. Genetics 147: 1783–1798. 9409836 10.1093/genetics/147.4.1783 PMC1208346

[bib59] Nelson C. Ambros V. Baehrecke E. H., 2014 *miR-14* regulates autophagy during developmental cell death by targeting *ip3-kinase 2*. Mol. Cell 56: 376–388. 25306920 10.1016/j.molcel.2014.09.011 PMC4252298

[bib60] Nohara L. L. Stanwood S. R. Omilusik K. D. Jefferies W. A., 2015 Tweeters, woofers and horns: the complex orchestration of calcium currents in T lymphocytes. Front. Immunol. 6: 234. 26052328 10.3389/fimmu.2015.00234 PMC4440397

[bib61] Ohno Y. Otaki J. M., 2015 Spontaneous long-range calcium waves in developing butterfly wings. BMC Dev. Biol. 15: 17. 25888365 10.1186/s12861-015-0067-8 PMC4445562

[bib62] Parker D. R., 1935 Locus of wy2 (formerly cxb). Drosoph. Inf. Serv. 4: 62.

[bib63] Parks A. L. Cook K. R. Belvin M. Dompe N. A. Fawcett R., 2004 Systematic generation of high-resolution deletion coverage of the *Drosophila melanogaster* genome. Nat. Genet. 36: 288–292. 14981519 10.1038/ng1312

[bib64] Ramos I. Wessel G. M., 2013 Calcium pathway machinery at fertilization in echinoderms. Cell Calcium 53: 16–23. 23218671 10.1016/j.ceca.2012.11.011 PMC4778076

[bib65] Ren N. Zhu C. M. Lee H. Adler P. N., 2005 Gene expression during *Drosophila* wing morphogenesis and differentiation. Genetics 171: 625–638. 15998724 10.1534/genetics.105.043687 PMC1456776

[bib66] Roderick H. L. Knollmann B. C., 2013 Inositol 1,4,5-trisphosphate receptors “exciting” players in cardiac excitation-contraction coupling? Circulation 128: 1273–1275. 23983251 10.1161/CIRCULATIONAHA.113.005157 PMC3885819

[bib67] Rodriguez A. D. Didiano D. Desplan C., 2012 Power tools for gene expression and clonal analysis in *Drosophila*. Nat. Methods 9: 47–55. 10.1038/nmeth.1800 PMC3574576 22205518

[bib68] Rorth P. Szabo K. Bailey A. Laverty T. Rehm J., 1998 Systematic gain-of-function genetics in *Drosophila*. Development 125: 1049–1057. 9463351 10.1242/dev.125.6.1049

[bib69] Ryder E. Ashburner M. Bautista-Llacer R. Drummond J. Webster J., 2007 The DrosDel deletion collection: a *Drosophila* genome-wide chromosomal deficiency resource. Genetics 177: 615–629. 17720900 10.1534/genetics.107.076216 PMC2013729

[bib70] Seeds A. M. Sandquist J. C. Spana E. P. York J. D., 2004 A molecular basis for inositol polyphosphate synthesis in *Drosophila melanogaster*. J. Biol. Chem. 279: 47222–47232. 15322119 10.1074/jbc.M408295200

[bib71] Shah S. Z. Zhao D. Khan S. H. Yang L., 2015 Regulatory mechanisms of endoplasmic reticulum resident IP3 receptors. J. Mol. Neurosci. 56: 938–948. 25859934 10.1007/s12031-015-0551-4

[bib72] St Pierre S. E. Ponting L. Stefancsik R. McQuilton P. Consortium F., 2014 FlyBase 102-advanced approaches to interrogating FlyBase. Nucleic Acids Res. 42: D780–D788. 24234449 10.1093/nar/gkt1092 PMC3964969

[bib73] Stapleton M. Liao G. C. Brokstein P. Hong L. Carninci P., 2002 The *Drosophila* gene collection: identification of putative full-length cDNAs for 70% of *D. melanogaster* genes. Genome Res. 12: 1294–1300. 12176937 10.1101/gr.269102 PMC186637

[bib74] Swarup S. Verheyen E. M., 2012 Wnt/wingless signaling in *Drosophila*. Cold Spring Harb. Perspect. Biol. 4: pii: a007930. 10.1101/cshperspect.a007930 PMC3367557 22535229

[bib75] Tamura K. Peterson D. Peterson N. Stecher G. Nei M., 2011 MEGA5: Molecular evolutionary genetics analysis using maximum likelihood, evolutionary distance, and maximum parsimony methods. Mol. Biol. Evol. 28: 2731–2739. 21546353 10.1093/molbev/msr121 PMC3203626

[bib76] Taylor J. Adler P. N., 2008 Cell rearrangement and cell division during the tissue level morphogenesis of evaginating *Drosophila* imaginal discs. Dev. Biol. 313: 739–751. 18082159 10.1016/j.ydbio.2007.11.009 PMC2258245

[bib77] Theibert A. B. Estevez V. A. Ferris C. D. Danoff S. K. Barrow R. K., 1991 Inositol 1,3,4,5-tetrakisphosphate and inositol hexakisphosphate receptor proteins - isolation and characterization from rat-brain. Proc. Natl. Acad. Sci. USA 88: 3165–3169. 1849645 10.1073/pnas.88.8.3165 PMC51406

[bib78] Thibault S. T. Singer M. A. Miyazaki W. Y. Milash B. Dompe N. A., 2004 A complementary transposon tool kit for *Drosophila melanogaster* using P and piggyBac. Nat. Genet. 36: 283–287. 14981521 10.1038/ng1314

[bib79] Venkatesh K. Hasan G., 1997 Disruption of the IP3 receptor gene of *Drosophila* affects larval metamorphosis and ecdysone release. Curr. Biol. 7: 500–509. 9273145 10.1016/s0960-9822(06)00221-1

[bib80] Venken K. J. T. Popodi E. Holtzman S. L. Schulze K. L. Park S., 2010 A Molecularly defined duplication set for the X Chromosome of *Drosophila melanogaster*. Genetics 186: 1111–1125. 20876565 10.1534/genetics.110.121285 PMC2998297

[bib81] Venkiteswaran G. Hasan G., 2009 Intracellular Ca2+ signaling and store-operated Ca2+ entry are required in *Drosophila* neurons for flight. Proc. Natl. Acad. Sci. USA 106: 10326–10331. 19515818 10.1073/pnas.0902982106 PMC2700899

[bib82] Vervloessem T. Yule D. I. Bultynck G. Parys J. B., 2015 The type 2 inositol 1,4,5-trisphosphate receptor, emerging functions for an intriguing Ca(2+)-release channel. Biochim. Biophys. Acta 1853: 1992–2005. 25499268 10.1016/j.bbamcr.2014.12.006 PMC4465056

[bib83] Wilkie A. O. M., 1994 The molecular-basis of genetic dominance. J. Med. Genet. 31: 89–98. 8182727 10.1136/jmg.31.2.89 PMC1049666

[bib84] Wong L. L. Adler P. N., 1993 Tissue polarity genes of *Drosophila* regulate the subcellular location for prehair initiation in pupal wing cells. J. Cell Biol. 123: 209–221. 8408199 10.1083/jcb.123.1.209 PMC2119819

[bib85] Wong R. Hadjiyanni I. Wei H. C. Polevoy G. McBride R., 2005 PIP2 hydrolysis and calcium release are required for cytokinesis in *Drosophila* spermatocytes. Curr. Biol. 15: 1401–1406. 16085493 10.1016/j.cub.2005.06.060

[bib86] Xia H. J. Yang G., 2005 Inositol 1,4,5-trisphosphate 3-kinases: functions and regulations. Cell Res. 15: 83–91. 15740635 10.1038/sj.cr.7290270

[bib87] Yang L. Meng F. Ma D. Xie W. Fang M., 2013 Bridging decapentaplegic and wingless signaling in *Drosophila* wings through repression of naked cuticle by Brinker. Development 140: 413–422. 23250215 10.1242/dev.082578

