# Supplementary material for: The wavy Mutation Maps to the Inositol 1,4,5-Trisphosphate 3-Kinase 2 (IP3K2) Gene of Drosophila and Interacts with IP3R to Affect Wing Development
Source: G3 (Bethesda). 2015 Nov 25;6(2):299–310. doi: 10.1534/g3.115.024307 (PMC4751550; doi:10.1534/g3.115.024307)
Supplement: Supporting Information [file supp_6_2_299__index.html]

The wavy Mutation Maps to the Inositol 1,4,5-Trisphosphate 3-Kinase 2 (IP3K2) Gene of Drosophila and Interacts with IP3R to Affect Wing Development — Supporting Information 

# The *wavy* Mutation Maps to the *Inositol 1,4,5-Trisphosphate 3-Kinase 2* (*IP3K2*) Gene of *Drosophila* and Interacts with *IP3R* to Affect Wing Development

## Supporting Information for Dean *et al.*, 2016

**Files in this Data Supplement:**

- File S1 - Fly media. (.pdf, 326 KB)
- Table S1 - PCR/sequencing primers. (.pdf, 328 KB)
- Table S2 - Wing scoresa of *nub*-GAL4 *Tub*-GAL80ts/+; RNAi-*IP3K2*/+ flies under expressing (29°C) and non-expressing (18°C) conditions. (.pdf, 433 KB)
- Table S3 - Testing for dominant modification of the *wy* phenotype by mutations in the IP3 signaling pathway genes *IP3K1*, *Ipk2*, and *Cama*. (.pdf, 438 KB)
