# Supplementary material for: The wavy Mutation Maps to the Inositol 1,4,5-Trisphosphate 3-Kinase 2 (IP3K2) Gene of Drosophila and Interacts with IP3R to Affect Wing Development
Source: G3 (Bethesda). 2015 Nov 25;6(2):299–310. doi: 10.1534/g3.115.024307 (PMC4751550; doi:10.1534/g3.115.024307)
Supplement: Supporting Information [file supp_g3.115.024307_FileS1.pdf]

File S1

Fly media

For every 100 ml of near-boiling distilled water, stir the following in sequentially:

11 g dextrose (Fisher Scientific)

1.5 g agar (Fisher Scientific)

5 g Baker's yeast (Red Star)

5.2 g commercial cornmeal

Take diet off of heat and blend to cool below 60°C. While blending, add 750 µl of propionic acid (Sigma) and 500 µl of 15% p-hydroxybenzoic acid methyl ester (Sigma), the latter dissolved in 95% ethanol. After dispensing into vials or bottles and allowing time for diet to congeal, supplement with pinches of Baker's yeast and cap.
