# Supplementary material for: The wavy Mutation Maps to the Inositol 1,4,5-Trisphosphate 3-Kinase 2 (IP3K2) Gene of Drosophila and Interacts with IP3R to Affect Wing Development
Source: G3 (Bethesda). 2015 Nov 25;6(2):299–310. doi: 10.1534/g3.115.024307 (PMC4751550; doi:10.1534/g3.115.024307)
Supplement: Supporting Information [file supp_g3.115.024307_TableS1.pdf]

Table S1 PCR/sequencing primers

Primer name	Sequence
IP3K2-1S	5'-CGC TAT CAG GTA TTG CCA CTG ATG CAC-3'
IP3K2-2AS	5'-AGC GTT GGC ATT GCT GGC GC-3'
IP3K2-2S	5'-GCG CCA GCA ATG CCA ACG CT-3'
IP3K2-3S	5'-CCA TGG GTT CAA CTG GCC GG-3'
IP3K2-3AS	5'-CCG GCC AGT TGA ACC CAT GG-3'
IP3K2-4S	5'-GCC CCG CTA CAT ACA GCG TTT G-3'
IP3K2-5AS	5'-GCC CCA AAC CTC CGT GTG GA-3'
