# Supplementary material for: The wavy Mutation Maps to the Inositol 1,4,5-Trisphosphate 3-Kinase 2 (IP3K2) Gene of Drosophila and Interacts with IP3R to Affect Wing Development
Source: G3 (Bethesda). 2015 Nov 25;6(2):299–310. doi: 10.1534/g3.115.024307 (PMC4751550; doi:10.1534/g3.115.024307)
Supplement: Supporting Information [file supp_g3.115.024307_TableS2.pdf]

TABLE S2 Wing scores[a] of *nub*-GAL4 *Tub*-GAL80^{ts}/+; RNAi-*IP3K2*/+ flies under expressing (29°C) and non-expressing (18°C) conditions

Incubation conditions[b]	n	% with each wing score				Average wing score
		0	1	2	3	
A. Controls						
29°C	87	10.3	0.0	63.3	26.4	2.1
18°C	69	100.0	0.0	0.0	0.0	0.0
B. 29°C to 18°C shift						
Emb	18	100.0	0.0	0.0	0.0	0.0
Larv	34	100.0	0.0	0.0	0.0	0.0
Wand	68	100.0	0.0	0.0	0.0	0.0
Pup	26	42.3	0.0	46.2	11.5	1.2*
Meta	67	29.9	10.4	32.8	26.9	1.5*
C. 18°C to 29°C shift						
Emb	37	5.4	0.0	67.6	27.0	2.1
Larv	95	10.5	4.2	74.8	10.5	1.8*
Wand	41	12.2	0.0	85.4	2.4	1.7*
Pup	20	0.0	75.0	25.0	0.0	1.1*
Meta	39	100.0	0.0	0.0	0.0	0.0

[a]See first section of Results text and Figure 2B-E for a detailed description of the scoring system.

[b]See Figure 4 caption for a detailed description of the experimental design and developmental staging codes.

*$p<0.05$ for the Fisher's exact tests comparing the marked experimental group to each of the two controls in Section A. (For all unmarked experimental groups, $p>0.05$ when tested against one of these two controls, and $p<<0.05$ when tested against the other control.)
