# Supplementary material for: The wavy Mutation Maps to the Inositol 1,4,5-Trisphosphate 3-Kinase 2 (IP3K2) Gene of Drosophila and Interacts with IP3R to Affect Wing Development
Source: G3 (Bethesda). 2015 Nov 25;6(2):299–310. doi: 10.1534/g3.115.024307 (PMC4751550; doi:10.1534/g3.115.024307)
Supplement: Supporting Information [file supp_g3.115.024307_TableS3.pdf]

TABLE S3 Testing for dominant modification of the *wy* phenotype by mutations in the IP_3 signaling pathway genes *IP3K1, Ipk2*, and *Cam*[a]

Genotype	n	% with each wing score				Average wing score
		0	1	2	3	
A. *IP_3 3-kinase 1* (*IP3K1*)[b]						
wy^2; *$IP3K1^{KG02192}$*/+	53	0.0	22.6	45.3	32.1	2.1*
wy^2; +/+	64	0.0	23.4	51.6	25.0	2.0
B. *Inositol polyphosphate kinase* (*Ipk2*)[c]						
wy^2; Df-*Ipk2*/+	41	0.0	2.4	70.7	26.8	2.2*
wy^2; +/+	38	0.0	5.3	52.6	42.1	2.4
C. *Calmodulin (Cam)*[d]						
wy^2; *Cam^{n339}*/+	83	0.0	24.1	66.3	9.6	1.8*
wy^2; +/+	64	0.0	12.5	73.4	14.1	2.0
wy^2; *Cam^7*/+	61	1.6	14.8	80.3	3.3	1.8*
wy^2; +/+	39	0.0	12.8	71.8	15.4	2.0

[a]See first section of Results text and Figure 2B-E for a detailed description of the scoring system.

[b]*w wy^2 f* females were crossed to *y^1*; *$IP3K1^{KG02192}$*/CyO; *ry^{506}* males and *w wy^2 f*/Y; *$IP3K1^{KG02192}$*/+ F1 males were collected. (This removed the CyO balancer from the crosses, allowing *wy* to be scored without obfuscation from the similar *Cy* trait.) These F1 males were crossed to *w wy^2 f* females, and *w wy^2 f*/Y; *$IP3K1^{KG02192}$*/+ F2 males were compared to their *w wy^2 f*/Y; +/+ male sibling controls. These F2 males were able to be distinguished because the *$IP3K1^{KG02192}$* P-insertion carries the *w^+* allele.

[c]*w wy^2 f* females were crossed to Df-*Ipk2*/SM6a, *y^1 w* males to, as discussed in [b] above, remove the SM6a-associated *Cy* marker from the cross scheme. *w wy^2 f*/Y; Df-*Ipk2*/+ F1 males were crossed to *w wy^2 f* females, and *w wy^2 f*/Y; Df-*Ipk2*/+ F2 males were compared to their *w wy^2 f*/Y; +/+ male sibling

controls. These F2 males were able to be distinguished because transposable elements associated with the Df-*Ipk2* deletion carry the w^+ allele (Ryder *et al.* 2007).

[d] *y^1 w; Act5C*-GAL4, *Cam^+*/CyO, *y^+* females (BL 4414) were crossed to either *y^1 w; Cam^{n339}*/CyO, *y^+* or *y^1 w; Cam^7*/CyO, *y^+* males. *y^1 w; Cam*/*Act5C*-GAL4, *Cam^+* F1 males were selected to, as discussed in [b] above, remove the CyO-associated *Cy* marker from the cross scheme. *w wy^2 f*/Y; *Cam*/*Act5C*-GAL4, *Cam^+* F1 males were crossed to *w wy^2 f* females, and *w wy^2 f*/Y; *Cam*/+ F2 males were compared to their *w wy^2 f*/Y; *Act5C*-GAL4, *Cam^+*/+ male sibling controls (for simplicity, controls represented as "*wy^2*; +/+" in the table). These F2 males were able to be distinguished because the *Act5C*-GAL4 construct carries a w^+ allele.

*p>0.10, Fisher's exact test vs. sibling controls in the row immediately below the marked row.
